# Triple-mode point-of-care diagnostics for high-risk human papillomavirus in urine

**DOI:** 10.1016/j.biosx.2025.100658

**Published:** 2025-07-07

**Authors:** Duc Anh Thai, Jing Liu, Angel Gutierrez Ruiz, Yuqian Zhang, Marina Walther-Antonio, Yuguang Liu

**Affiliations:** a Department of Physiology and Biomedical Engineering, Mayo Clinic, Rochester, MN, 55905, USA; b Microbiome Program, Center for Individualized Medicine, Mayo Clinic, Rochester, MN, 55905, USA; c Department of Chemistry and Biochemistry, The University of Texas at El Paso, El Paso, TX, 79968, USA; d Department of Obstetrics and Gynecology, Mayo Clinic, Rochester, MN, 55905, USA; e Department of Surgery, Mayo Clinic, Rochester, MN, 55905, USA; f Department of Immunology, Mayo Clinic, Rochester, MN, 55905, USA

**Keywords:** Human papillomavirus, Cervical cancer, Loop-mediated isothermal amplification, Point-of-care, Triple-mode detection

## Abstract

Human papillomavirus (HPV) screening is crucial for early diagnosis and prevention of cervical cancer, yet fast and convenient HPV detection remains challenging, especially in resource-limited areas. Herein, we developed a nucleic acid test named SSMG-LAMP, which combined loop-mediated isothermal amplification (LAMP) with an engineered DNA indicator (SYBR Safe – Malachite Green) for the point-of-care diagnosis of high-risk HPV strains (HPV 16 and 18) in urine. The assay can be completed within 45 min, including DNA extraction, SSMG-LAMP reaction, and signal readout using a simple, portable system. This system enabled the triple-mode detection of DNA targets using colorimetry, fluorometry, and electrochemistry, and can detect as low as 10 copies μL^−1^ HPV DNA. As a preliminary validation, we used SSMG-LAMP for a blind test of 16 clinical urine samples to detect HPV 16 and 18, and results showed a sensitivity of >80 % and specificity of up to 96.2 %, with a 95 % confidence interval. This triple-mode HPV detection strategy holds potential for point-of-care cervical cancer screening in low-resource settings.

## 1. Introduction

Cervical cancer is the fourth most common gynecological malignancy. In 2022 alone, there were approximately 660,000 new cases and 350,000 deaths worldwide (Bray et al., 2024). However, the risk of developing cervical cancer can be greatly reduced by routinely screening for high-risk human papillomavirus (HPV) – a common sexually transmitted virus that causes 99 % of cervical cancer cases (Cohen et al., 2019). The most common high-risk HPV types are 16 and 18, accounting for over 70 % of invasive cervical cancer cases (de Sanjose et al., 2010). High-risk HPV strains can cause abnormal tissue growth (e.g., warts), therefore persistent infection can eventually lead to the formation of precancerous and cancerous legions in the cervix. Direct nucleic acid test (NAT) and nucleic acid amplification test (NAAT) are among the most effective methods for high-risk HPV screening (Mou et al., 2021). In direct NAT, the presence of high-risk HPV DNA or RNA in cells can be rapidly detected based on, for example, fluorescent in-situ hybridization, but the technique is generally limited in sensitivity (Ratnam et al., 2011). In contrast, NAAT can amplify specific genes and thus offers increased sensitivity. Polymerase chain reaction (PCR) remains the gold standard for HPV screening, and detects high-risk HPV infection based on specific genes, for example, E6 or E7 gene sequences, which are typically present in high-risk subtypes of HPV (Okodo et al., 2018; Weng et al., 2021). However, these methods often require skilled technicians, multistep procedures, and bulky, specialized equipment in centralized labs, making them difficult to access in rural areas or low-resource settings. Because of the inequalities in access to timely diagnosis and treatment along with socioeconomic determinants, >90 % of cervical cancer-related deaths occur in low-income areas such as sub-Saharan Africa, southeast Asia, and central America (Tewari, 2025). Therefore, a rapid, simple and inexpensive point-of-care HPV screening test is ideal for preventing cervical cancer in areas with limited healthcare resources.

Loop-mediated isothermal amplification (LAMP) has been increasingly adopted for point-of-care testing (POCT) of infectious diseases due to its simplicity, cost-effectiveness, and rapidity as well as high sensitivity and specificity (Bai et al., 2024; Sun et al., 2024; Wang et al., 2022). Unlike PCR, LAMP operates at a constant temperature (e.g., 65 °C) without a need for thermal cycling but offers comparable sensitivity within a shorter time (30–60 min) (Bartosik et al., 2024). Moreover, LAMP can be easily coupled with several detection methods, such as colorimetry, fluorometry, and electrochemistry, that are suitable for POCT applications in remote areas (Chandrasekaran et al., 2022; Chen et al., 2018; Dao Thi et al., 2020; Izadi et al., 2021). Among various detection mechanisms, a straightforward strategy is to use intercalating dyes - hydrophobic molecules that bind to DNA and alter its structure, for example, ethidium bromide, to detect DNA produced in LAMP assays. These intercalating dyes, however, often cause mutations in DNA and increase the risk of cancer. Less mutagenic and non-mutagenic options include, for instance, SYBR Green I and SYBR Safe (SS), sensitive nucleic acid stains, which are often used in LAMP and emits significant fluorescence under UV or blue light (Ding et al., 2015; Dixit et al., 2018; Ruang-areerate et al., 2022). While these dyes can also be used in elevated concentrations to visually detect color changes under natural light, high concentrations of these molecules can inhibit LAMP reaction. As an alternative, chromogenic intercalators can enable the visual detection of DNA based on color changes under ambient light. For example, a cationic dye, malachite green (MG), preferentially binds to the base pairs of double-stranded DNA (dsDNA) *via* aromatic ring stacking interactions, which causes a shift in its absorbance spectrum toward a longer wavelength (Li et al., 2024; Scott et al., 2020). However, the shift in the spectral wavelength is often narrow (from blue toward pale blue or blue-green), which can cause ambiguous result interpretations and false result readout, especially in weak-positive reactions.

To leverage the unique merits of distinct types of dyes, some efforts have been focused on coupling the use of different dyes for DNA detection in LAMP assays. For example, Zhang et al. combined cresol red (pH indicator) and hydroxyl naphthol blue (Mg^2+^ indicator) in a LAMP reaction for enhanced colorimetric detection (Zhang et al., 2022). This was because, during DNA amplification, as the nucleotides were incorporated into the growing strands, protons were released, causing a decrease in pH and, thus, a color change; meanwhile, as DNA was amplified, the concentration of Mg^2+^ ions in the solution decreased significantly, causing additional color change. Likewise, Ding et al. combined hydroxyl naphthol blue and SYBR Green I to better distinguish weak-positive and negative results and enable DNA detection under fluorescent excitation (Ding et al., 2015). Inspired by these strategies, here we coupled SS and MG for LAMP assays in low-resource settings, named SSMG-LAMP, to improve the quantification of amplified DNA. There are several advantages of this work. First, based on the color evolution principle, the blending of SS and MG can expand the shift in absorbance spectrum, leading to clear color differences in weak-positive and negative results under natural light. Second, the synergistic effect of fluorescent and chromogenic DNA intercalating mechanisms can, by nature, produce an orange/green fluorescence in the absence/presence of DNA, making it easier to distinguish background noise and weak-positive results under fluorescent excitation. Third, as MG possesses a positive charge and aromatic rings, it can interact with double-stranded DNA and undergo redox reactions in the electrodes and generate electrical signals (Hu et al., 2006), offering electrochemical detection as a third detection mode in the LAMP assay. While most LAMP assays adopt a single mode of readout, mostly colorimetric or fluorescence, multiple modes of signal output can improve the accuracy of the assay and provide versatile formats that are most suited to specific scenarios (Dong et al., 2024). In this work, a proof-of-concept assay was developed to detect high-risk HPV in clinical urine samples within 45 min, with a limit of detection of 10 copies μL^−1^. This strategy is simple, rapid and effective, suitable for field-deployable HPV diagnosis for cervical cancer screening in low-resource settings.

## 2. Experimental section

### 2.1. Materials

SYBR Safe (SS) and Ambion nuclease-free water were purchased from Invitrogen Thermo Fisher Scientific (Carlsbad, CA). Malachite green (MG) was supplied by VWR Life Science AMRESCO (Rochester, NY). Deoxynucleotide (dNTPs) solution mix (10 mM each dNTP) and LAMP kit including 8000 U mL^−1^ *Bst* 2.0 WarmStart DNA Polymerase (M0538), 10 × isothermal amplification buffer, and 100 mM MgSO_4_ were purchased from New England Biolabs (Ipswich, MA). High Sensitivity D1000 ScreenTape assay and reagents were provided by Agilent Technologies (Santa Clara, CA). HPV 16 (ATCC 45113D), HPV 18 (ATCC 45152D), and HPV 11 (ATCC 45151D) purified plasmid DNA were supplied by American Type Culture Collection (Manassas, VA). Quick-DNA/RNA Viral Kit was purchased from Zymo Research (Irvine, CA). LAMP primer sets were designed based on the *E6* gene sequence of HPV using the Primer Explorer V5 software (Eiken Chemical Co. Ltd, Tokyo, Japan). The primers were synthesized and purified by Integrated DNA Technologies (Coralville, IA). All the primer sequences are listed in Table S1.

### 2.2. SSMG-LAMP assay

A 25 μL LAMP reaction was performed to detect HPV 16 and 18. LAMP reaction consisted of 1 × isothermal amplification buffer, 8 mM MgSO_4_, 1.4 mM of each dNTP, 0.2 μM of F3/B3 primers, 1.6 μM of FIP/BIP primers, 0.4 μM of LF/LB primers, 8 U of *Bst* 2.0 WarmStart DNA Polymerase, and DNA template. The reaction was incubated using a portable mini16 thermal cycler (miniPCR, Cambridge, MA) and the protocol operation was controlled by the smartphone miniPCR APP. 2 μL of SSMG was added to the tube lid before amplification and then reacted with LAMP products after incubation. Various concentrations of SS (1, 10, 25, and 50 ×) and MG (10, 25, 50, and 100 μM) were tested to determine the optimized concentration of SSMG. The effect of different incubation times (20–50 min) and temperatures (60–65 °C) was also assessed to optimize the SSMG-LAMP assay.

### 2.3. Electrophoresis analysis

Capillary electrophoresis was employed to verify the LAMP reaction product using the 4150 TapeStation (Agilent Technologies). Each sample (containing 1 μL of LAMP product and 3 μL of buffer) was loaded into the High Sensitivity D1000 ScreenTape. The results were analyzed by TapeStation Analysis Software.

### 2.4. Colorimetric analysis

After incubation, the color change of the reaction mixture was first evaluated visually and then recorded using a smartphone camera. The RGB value was processed using ImageJ software, and the color difference between negative and positive samples was obtained using the CIEDE2000 (ΔE) calculator. ΔE uses CIELAB coordinates that bring color differences closer to what the human eye perceives (Luo et al., 2001). The UV–Vis absorbance was recorded in a 400–700 nm range using the CLARIOstar Plus microplate reader (BMG LABTECH, Cary, NC).

### 2.5. Fluorescent analysis

The fluorescence change was monitored under the blue LED light using a handheld P51 molecular fluorescence viewer (miniPCR, Cambridge, MA). The fluorescence emission was measured in a range of 500–700 nm with an excitation wavelength of 470 nm using the CLARIOstar Plus microplate reader.

### 2.6. Electrochemical analysis

The electrochemical assay was performed using electrode chemical sensors with bare gold electrode microfabricated as in our previous work (Telic Company, Santa Clarita, CA). The electrochemical signals were measured by a CHI660E electrochemical workstation in a Picoamp Booster and Faraday Cage (CHI200B, CH Instruments, Inc., Austin, TX), following our previously developed method (Zhang et al., 2024). 20 μL of SSMG-LAMP product was dropped onto the screen-printed electrode, covering all the electrode area, to conduct electrochemical measurements. Cyclic voltammetry was scanned in the potential range from – 1.5 V to 1.2 V at various scan rates (0.02–0.2 V s^−1^).

### 2.7. Sensitivity, specificity, and stability tests

SSMG-LAMP assays were conducted with 10-fold serial dilution of DNA templates and optimal conditions were used to evaluate the detection sensitivity. To test the specificity, an SSMG-LAMP assay was performed with each designed primer set to specifically identify HPV 16 and 18. The as-prepared SSMG was stored at room temperature in the dark, and the performance of the SSMG-LAMP assay was evaluated weekly to determine the feasibility of long-term storage.

### 2.8. Clinical sample collection and processing

Urine samples (n = 16) used in this study were collected under the Institutional Review Board (IRB) 18–005528. All patients in this study provided written consent under IRB 18–005528. Inclusion criteria for enrollment consisted of patients being tested for HPV 16 and/or HPV 18 for clinical diagnosis at Mayo Clinic, Rochester, MN. Vulnerable patients or patients unable to provide informed consent were excluded. The urine sample was collected by the patient (urine in a standard clinical vial) only identified by a Subject ID, time, and date. Urine samples were placed in research collection boxes, retrieved by the research team and stored in the laboratory at −80 °C until processing. Viral DNA was extracted from clinical urine samples using Quick-DNA/RNA Viral Kit, following Zymo Research’s instructions.

### 2.9. Statistical analysis

GraphPad Prism 10 software was used for graphical representation and statistical analysis. Data were presented as mean ± standard deviation of three to six replicates. Statistical significance (*p* < 0.05) was determined using a T-test and one-way ANOVA.

## 3. Results and discussion

### 3.1. Establishment of SSMG-LAMP

We employed LAMP that uses a strand-displacing DNA polymerase and a set of specifically designed primers (Table S1) that can recognize a total of eight distinct regions in the target sequences to isothermally amplify a specific HPV DNA. The reaction is initiated as the inner primers (FIP/BIP) bind to the target sequence and start DNA synthesis, displacing the original DNA strand by the DNA polymerase with strand displacement activity. The outer primers (F3/B3) then anneal to the displaced strand, and their extension creates stem-loop DNA structures. These stem-loop structures serve as templates for subsequent amplification rounds, leading to exponential amplification of the target DNA. The loop primers (LF/LB) then bind to these stem-loop structures, facilitating additional rounds of amplification to speed up the reaction (Notomi et al., 2000). The reaction continues in a cyclic manner, which produces a large amount of complex and variable DNA structures, appearing as ladder-like bands of different lengths in electrophoresis analysis (Fig. S1). To enable visualization and clear distinction between positive signals and background noises in LAMP reaction under natural light, we coupled two DNA intercalators: fluorescent indicator – SS and chromogenic indicator – MG. SS molecules can insert themselves between the bases of the DNA double helix, producing green fluorescence under blue light excitation. As DNA amplifies in LAMP reaction, an increased number of SS molecules bind to DNA, leading to a remarkable increase in fluorescent signal. MG binds to base pairs of dsDNA molecules through aromatic ring stacking interactions, causing a change in its color and a shift in its absorption spectrum.

**Fig. 1.**
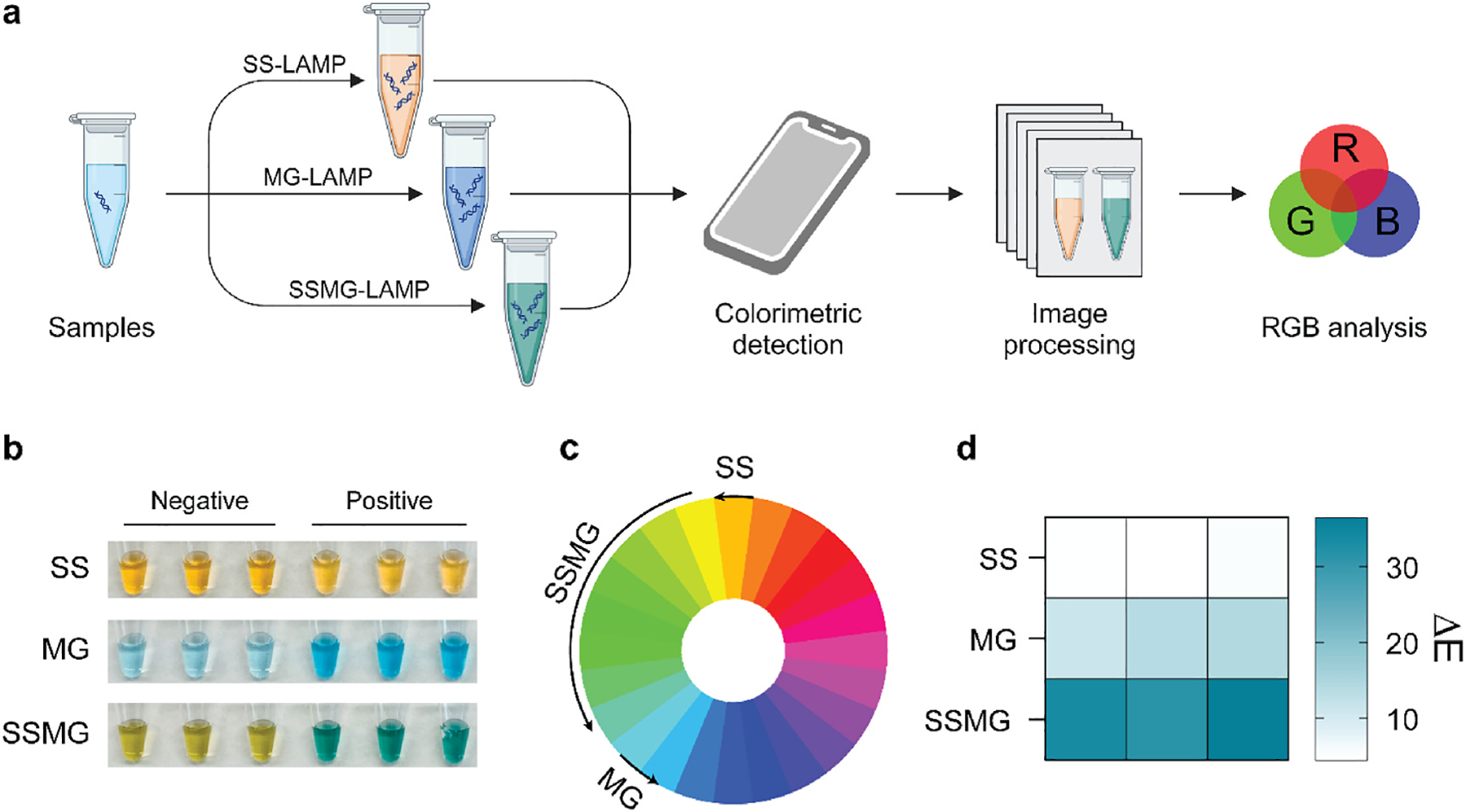
Overall scheme of colorimetric detection in LAMP. (a) Experimental workflow of LAMP visualization. (b) Representative image of colorimetric detection in LAMP using SS, MG and SSMG. (c) Spectrum range of color variation produced by SS, MG and SSMG. (d) Analysis of color differences (ΔE) between negative and positive samples using different indicators. SS: SYBR Safe, MG: malachite green. Negative control with no DNA template and Positive sample with 1200 copies μL^−1^ of HPV 16 DNA. The experiment was performed in three replicates.

To determine the effect of different indicators in LAMP reactions, we amplified 1200 copies μL^−1^ synthetic HPV 16 DNA in each tube for 30 min and added SS, MG and SSMG respectively. The colorimetric results were first detected *via* the naked eye and then recorded by a smartphone camera, and the RGB intensity in the images was further analyzed in the ΔE calculator (Fig. 1a). Under the ambient light, the use of SS led to a marginal difference in color in the HPV 16+ sample and the negative control. When MG was used as an indicator, the HPV 16+ sample turned blue while the negative control remained pale blue. In both cases, the insignificant color shift can cause ambiguity in result interpretations, especially when the tests are performed on physiological fluids (e.g., urine, saliva) by minimally trained users without image analysis tools. However, when SS and MG were coupled, a significant color shift from yellow to green was achieved, which makes the results easily distinguishable by the naked eye (Fig. 1b). Due to the color evolution principle, the blending of SS and MG expanded the shift in absorbance spectrum, leading to clear color differences under ambient light and offering significant color contrast between the HPV 16+ sample and the negative control (Fig. 1c). We then performed RGB analysis in CIELAB color space as it can precisely measure the color differences in a device-independent manner. The results confirmed that SS showed no distinction in color difference (ΔE) between the HPV 16+ sample and the negative control; MG showed an intermediate level of color difference but not as significant as SSMG (Fig. 1d).

Because of the synergistic effect of fluorescent (SS) and chromogenic (MG) DNA intercalating mechanism, the SSMG-LAMP assay enabled color changes under both ambient light and blue light excitation (Fig. S2). The conventional single-color fluorescence system often provides limited distinction between the weak-positive and negative samples. Our dual-color fluorescence model can overcome this limitation to easily indicate weak-positive reactions based on distinguishable fluorescent color changes. Moreover, this method can avoid presetting the cutoff values in every test to differentiate the reactions, especially in point-of-care settings. We also evaluated the use of other DNA intercalating dyes of SYBR family (Invitrogen Thermo Fisher Scientific) such as SYBR Green I and SYBR Gold in our system (Fig. S3). Although all SYBR dyes, in theory, can work in this assay, we chose SS because of its several unique advantages. Overall, SS can provide enhanced signals in both colorimetric and fluorescent detections (Fig. S3a and b). This type of DNA intercalating dye can be stored at room temperature, which critically benefits point-of-care applications in low-resource settings. SYBR Green I and SYBR Gold, however, require −20 °C storage condition. Moreover, SS is non-hazardous, environmental-friendly and is the most affordable in the SYBR series. A detailed comparison is shown in Fig. S3c.

**Fig. 2.**
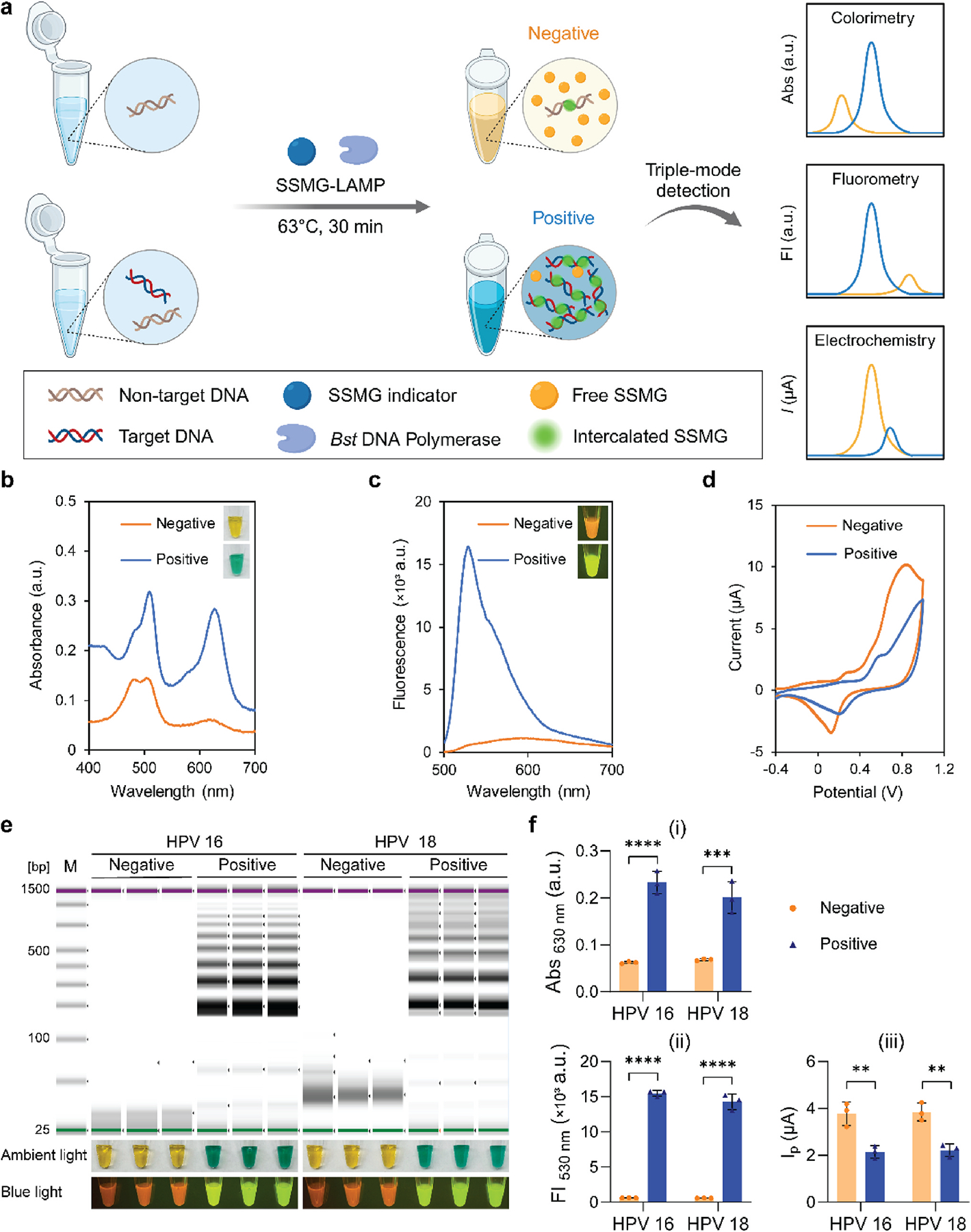
Principle of SSMG-LAMP assay for high-risk HPV detection. (a) Schematic diagram triple-mode detection in SSMG-LAMP. (b) UV–Vis absorption, (c) fluorescence emission, and (d) cyclic voltammetry plots of HPV + samples and negative controls. (e) Electrophoresis analysis, colorimetric visualization, and fluorescence detection of HPV 16 and 18. (f) Peak value of absorbance (i), fluorescence (ii), and current (iii) of negative and positive samples for colorimetric, fluorescent, and electrochemical detection of HPV 16 and 18, respectively. Negative control with no DNA template and Positive sample with 1200 (HPV 16) or 1000 (HPV 18) copies μL^−1^ of DNA template. M: DNA ladder, **p ≤ 0.01, ***p ≤ 0.001, ****p ≤ 0.0001. The experiment was performed in three replicates.

In addition, because the DNA amplicons and electroactive intercalated molecules can electrostatically interact and generate measurable electrical current on the surface of electrodes (Olabarria et al., 2020; Rioboó-Legaspi et al., 2024), we incorporated electrochemical sensing and established a triple-mode detection strategy in the SSMG-LAMP reaction to detect high-risk HPV strains through colorimetric, fluorescent, and electrochemical signals (Fig. 2a). In colorimetric measurement, the negative control presented yellow, while the HPV + sample was emerald green, with the absorption peak at around 510 nm and 630 nm, respectively (Fig. 2b). Fluorescence was observed under the blue light excitation (ʎ_ex_ = 470 nm), presenting a strong green emission in the HPV + sample and a weak orange emission in the negative control. While the fluorescence from the sample can be viewed by the naked eye in a handheld P51 molecular fluorescence viewer, the emission spectrum confirmed the significant difference in fluorescence intensity (ʎ_em_ = 530 nm) between the HPV + sample and the negative control (Fig. 2c). For electrochemical analysis, we investigated the effect of different scan rates (0.02–0.2 V s^−1^) on peak potential and current in cyclic voltammetry (Fig. S4). The scan rate of 0.1 V s^−1^ was chosen for subsequent experiments because the desired Faradaic current can be easily distinguished from the background. The plots in Fig. 2d showed a shift of peak potential in a positive direction and a decrease of peak current in the presence of target DNA. The intercalation of SSMG into DNA amplicons caused a response of lower peak current compared to the free-floating indicator in the negative control. In the redox reaction, the free electroactive molecules diffuse in the solution, transfer electrons to the electrode, and generate an electrical current. However, when electroactive molecules intercalate into dsDNA, their electron transfer reaction is hindered, resulting in a decrease in the electrochemical signal (Olabarria et al., 2020).

We designed the LAMP primers for HPV 16 and 18, the most common high-risk HPV subtypes determined by the *E6* gene sequence (Table S1). Prior to optimizing varied parameters in the SSMG-LAMP assay, we validated the quality of the LAMP-amplified HPV DNA using Tapestation. We used 1200 and 1000 copies μL^−1^ of synthetic HPV 16 and 18 DNA, respectively, in these validation tests, the results displayed clear bands (Fig. 2e), indicating the effectiveness of the primers and the LAMP reaction. The performance of the SSMG-LAMP assay was further evaluated using triple-signal measurement. As shown in Fig. 2f, all three outputs (colorimetry, fluorometry, and electrochemistry) successfully distinguished between HPV + samples and negative controls. This demonstrated the overall feasibility of using this detection mechanism in this SSMG-LAMP assay based on specific scenarios and environments.

### 3.2. Optimization of SSMG-LAMP

**Fig. 3.**
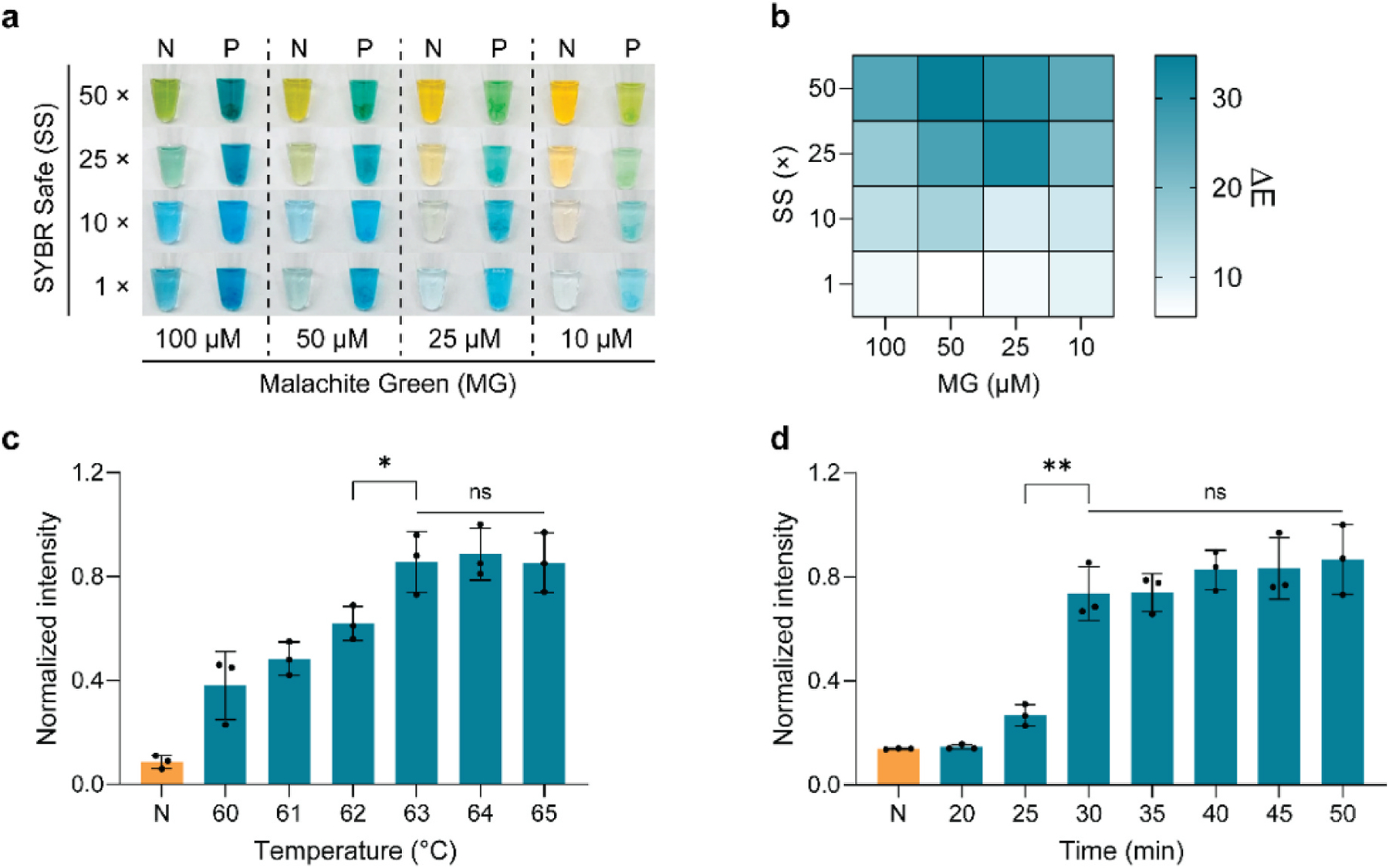
Optimization of SSMG-LAMP assay. (a) The effect of different SYBR Safe (SS) and malachite green (MG) concentrations on naked-eye observation. (b) Heat map of chromatic contrast between positive and negative samples with different concentrations of SS and MG. (c,d) Effects of reaction temperature (c) and time (d) on the performance of SSMG-LAMP. Color intensities were normalized against the maximum value. N: Negative control with no DNA template, P: Positive sample with 1200 copies μL^−1^ of HPV 16 DNA. ns: *p* > 0.05, *p ≤ 0.05, **p ≤ 0.01. The experiment was performed in three replicates.

Developing the SSMG-LAMP assay for point-of-care testing requires several key considerations. First, while the coupling of SS and MG can expand the shift in the absorbance spectrum, varied concentrations of each of the molecules present in a sample can lead to different colorimetric outcomes. Meanwhile, high concentrations of indicators can inhibit LAMP processes as these molecules can bind to DNA during the initial stages of the reaction. Second, it is ideal to perform these tests with lower temperatures within a shorter timeframe. To ensure the best outcome, we optimized varied parameters in the assay including the concentrations of SS and MG, reaction temperature, and reaction time. Results showed that the color change is strongly dependent on the concentration of these indicators. When SS concentration increased from 1 × to 50 ×, the color of the negative control shifted from blue to yellow, while the color of the HPV 16+ sample shifted from blue to green. Meanwhile, the increase in MG concentration (10–50 μM) increased the color difference between negative controls and HPV 16+ samples. However, the further elevated concentration of MG (100 μM) in the mixture limited the color transition in negative controls (Fig. 3a). According to the RGB analysis in CIELAB color space, the most significant chromatic contrast was achieved when the concentration of SS and MG was 50 × and 50 μM, respectively (Fig. 3b). Coupling the use of SS and MG at the optimized concentrations can lead to significant color changes that can be easily determined by the naked eye, which offers a straightforward distinction between weak-positive and negative samples. The assay was also optimized for different temperatures and times. Overall, an increase in temperature (60–65 °C) led to a strong readout, but the color intensity did not change significantly if the temperature was at least 63 °C. Therefore, we used 63 °C for subsequent experiments (Fig. 3c). Also, results showed that the LAMP readout was significantly enhanced after 30 min of reaction. Reaction beyond 30 min had negligible effects on the SSMG-LAMP outcome (Fig. 3d). The optimization study was then conducted with different concentrations of LAMP primers, in which a set of 0.2 μM of F3/B3 primers, 1.6 μM of FIP/BIP primers, and 0.4 μM of LF/LB primers was determined the optimal concentration for the SSMG-LAMP assay since it provided strong signal but low background noise (Fig. S5). These conditions were thus chosen for subsequent experiments.

### 3.3. Performance of SSMG-LAMP

**Fig. 4.**
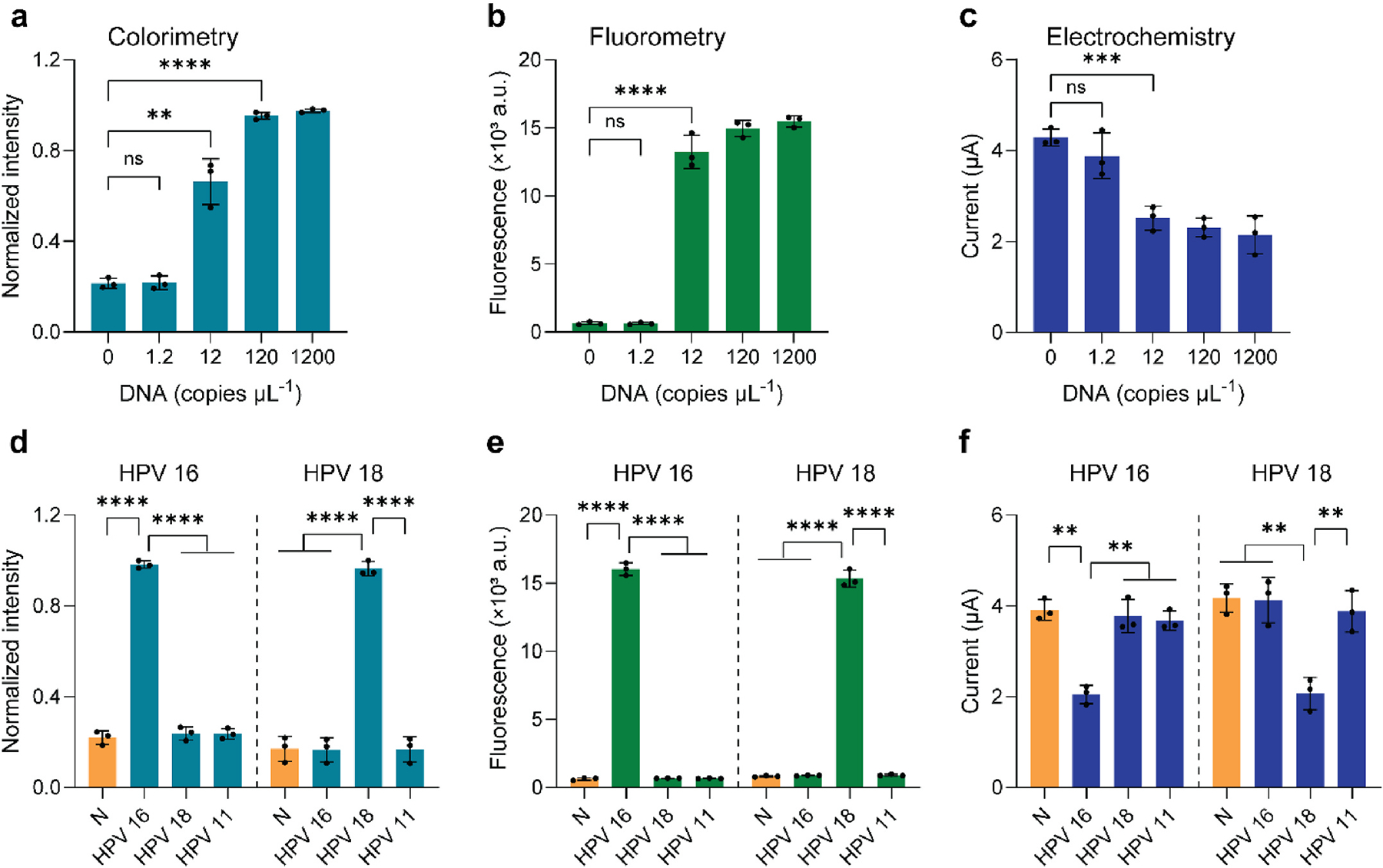
Sensitivity and specificity tests of the SSMG-LAMP assay. (a,b,c) The sensitivity test of SSMG-LAMP assay for colorimetric (a), fluorescent (b), and electrochemical (c) detection of HPV 16. (d,e,f) The specificity evaluation of SSMG-LAMP assay for colorimetric (d), fluorescent (e), and electrochemical (f) detection of HPV 16 and 18. N: Negative control with no DNA template, ns: *p* > 0.05, **p ≤ 0.01, ***p ≤ 0.001, ****p ≤ 0.0001. The experiment was performed in three replicates.

Using the optimized experimental conditions, we investigated the detection sensitivity of the SSMG-LAMP toward HPV 16 and 18 targets. These samples were analyzed using the triple-mode detection system. The HPV 16 detection in SSMG-LAMP achieved a sensitivity of 12 copies μL^−1^ for colorimetric, fluorescent, and electrochemical detections (Fig. 4a,b,c), while as low as 10 copies μL^−1^ HPV 18 were detected using these methods (Fig. S6). Detection limit in LAMP assays that use a single chromogenic DNA intercalator, such as MG, was relatively higher, 430 copies μL^−1^ for colorimetric detection and 22 copies μL^−1^ for electrochemical measurement (Rioboó-Legaspi et al., 2024). The detection limit in fluorescent mode is comparable with other dual fluorescence systems that achieved a detection sensitivity of 5–20 copies μL^−1^ (Ding et al. 2015, 2024). Our results indicate that coupling SS and MG improved the detection sensitivity of LAMP reactions, especially for colorimetric and electrochemical detection. Of note, this works as an advantage of this multi-mode detection system, allowing the users to adopt a detection strategy most suited to specific scenarios and environments.

Next, we examined the specificity of the assay as different subtypes of HPV, especially high-risk and low-risk strains. We chose HPV 11 as a common low-risk HPV strain to determine the specificity of this assay; this low-risk strain is linked to genital warts but is rarely associated with cancers (de Sanjose et al., 2010). For HPV 16 and 18 targets, the results showed significant colorimetric and fluorescence intensities, while the signals for the other targets remained close to the negative control (Fig. 4d and e). In electrochemical sensing, a decrease in the peak current indicated the presence of HPV 16 and 18 target DNA, while the signal for other interferences remained in the same range as in the negative control (Fig. 4f). SSMG molecules intercalated into the DNA amplicons produced from the HPV 16+ and 18+ LAMP reaction, leading to the significant reduction of free electroactive molecules, therefore resulting in a decrease of the peak current. Otherwise, low amounts of unamplified DNA from interfered targets did not change the electrochemical signal. These results showed that this triple-mode detection method is not only highly selective to high-risk HPV versus low-risk HPV 11 but can also distinguish between high-risk subtypes HPV 16/18, especially when these two strains share 60–70 % similarity in their genomic nucleotides (Harari et al., 2014).

For point-of-care testing in resource-limited settings, it is ideal to store reagents for the long term without refrigeration. To understand the stability of the SSMG indicator at room temperature over time, we performed stability tests over 4 weeks and assessed the effectiveness of the SSMG weekly. Over 4 weeks, the colorimetric, fluorescent, and electrochemical signal of SSMG-LAMP did not significantly decrease compared with the freshly prepared reaction (Fig. S7). Therefore, this reagent has the potential to be stored at room temperature for the long term, making the SSMG-LAMP excellent for field-deployable and point-of-care high-risk HPV screening in remote and resource-limited areas.

### 3.4. HPV detection in clinical samples

**Fig. 5.**
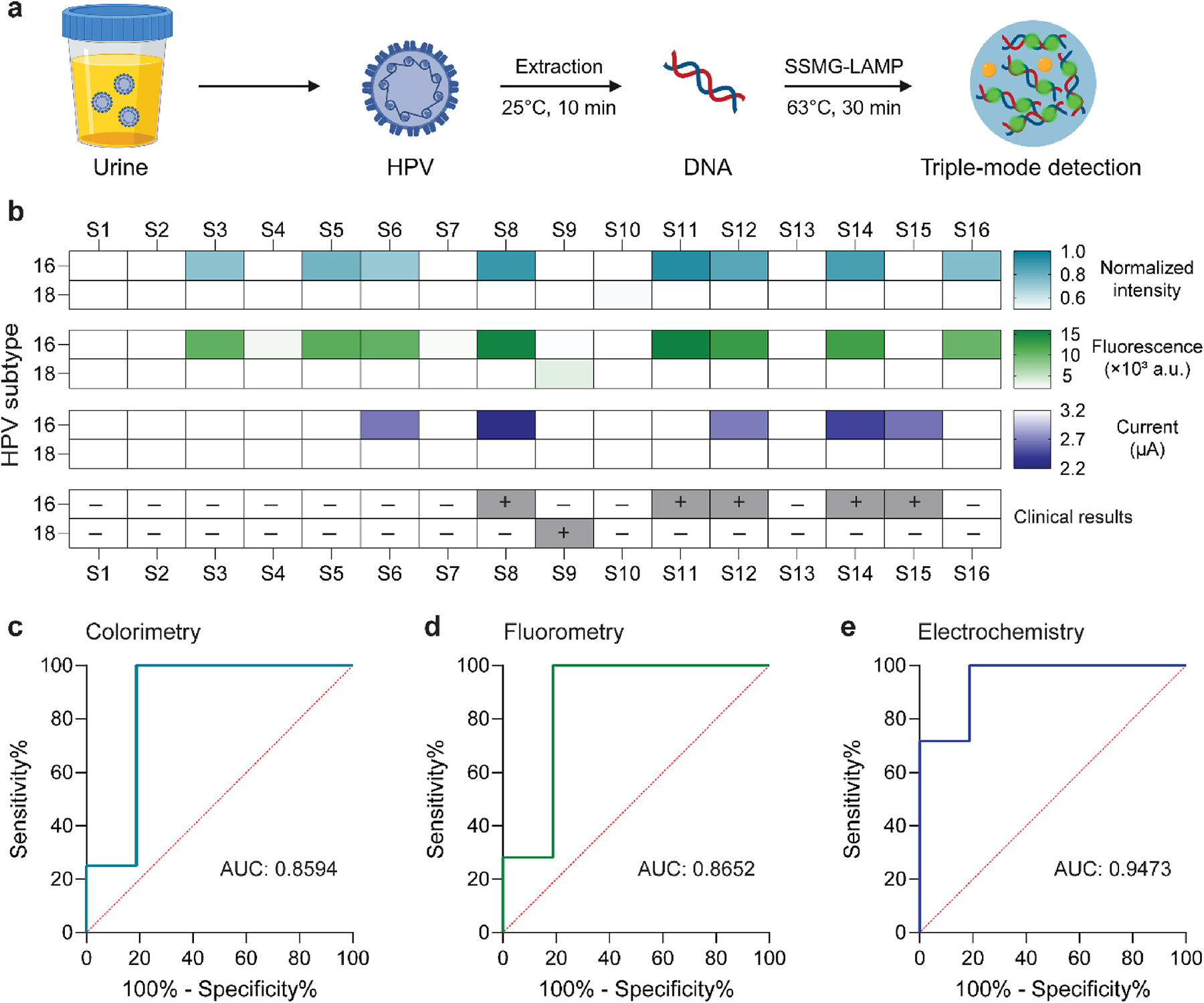
Detection of HPV 16 and 18 in clinical urine samples. (a) A scheme showing the clinical detection procedure. (b) Comparison of colorimetry, fluorometry, and electrochemistry methods and clinical results for HPV 16 and 18 detections from 16 urine samples. (c,d,e) The receiver operating characteristic (ROC) curves of colorimetric (c), fluorescent (d), and electrochemical (e) SSMG-LAMP in detecting clinical samples. The experiment was performed in six replicates.

Lastly, as a proof-of-concept, we used the SSMG-LAMP assay to detect high-risk HPV 16 and 18 strains in clinical urine samples collected and stored at Mayo Clinic (Rochester, MN). To ensure the reliability of the test and minimize subjectivity, we performed a blind test on 16 clinical urine samples. Prior to the experiments, the frozen urine samples were thawed at room temperature and gently vortexed to restore its homogeneity. First, DNA was extracted from 200 μL urine samples and suspended in 50 μL DNase/RNase-free water, following the manufacturer’s instructions. Then, the SSMG-LAMP assay was performed to detect HPV 16 and 18. After the SSMG-LAMP reaction, colorimetric, fluorescent and electrochemical detection were performed respectively. The entire workflow, including urinary DNA extraction, SSMG-LAMP reaction, and readout, was completed within 45 min (Fig. 5a).

The heatmap in Fig. 5b summarizes the results of HPV 16/18 detection using colorimetry, fluorometry, and electrochemistry, along with the clinically confirmed results by PCR. In the SSMG-LAMP assay, colorimetric and fluorescent readouts were in concordance with 4 out of the 5 clinically confirmed HPV 16+ samples and 7 out of the 11 clinically confirmed HPV 16− samples. In other words, 1 out of 5 samples returned false negative, and 4 out of 11 samples returned false positive. In electrochemical detection, results were in concordance with 4 out of the 5 clinically confirmed HPV 16+ samples, but 10 out of the 11 clinically confirmed negatives, leading to only 1 false positive result. Among all 16 urine samples, there was only 1 clinically confirmed HPV 18+ sample. No false positive result was observed in HPV 18 tests for all three methods. Nevertheless, only the fluorescent method detected the presence of HPV 18+ in the only clinically confirmed case, which showed the advantage of detecting a weak-positive that would otherwise be missed. Common causes for false negative results include DNA degradation (e.g., freeze and thaw) in specific samples (Peng et al., 2024) or low viral load that is beyond the limit of detection of this assay. On the other hand, false positives can sometimes be attributed to the presence of other clinically confirmed HPV subtypes in specific urine samples. For example, samples S3, S6, and S16 returned as false positive in HPV 16 detection (Table S2). Possible causes include the presence of HPV subtypes that are most closely related (genetically similar) to HPV 16 in the samples, such as HPV types 31, 33, and/or 52, which belong to the alpha-9 species group (Nelson and Mirabello, 2023). Contamination during sample handling is another common cause of false positive results.

We constructed the receiver operating characteristic (ROC) curve to systematically analyze the diagnostic performance of SSMG-LAMP for both HPV subtypes in all 16 urine samples, with a 95 % confidence interval (CI). The area under the curve (AUC), generated from the ROC curve, of the colorimetric, fluorescent, and electrochemical tests were 0.8594 (95 % CI: 0.7536–0.9651), 0.8652 (95 % CI: 0.7632–0.9673), and 0.9473 (95 % CI: 0.8981–0.9964), respectively (Fig. 5c,d,e). Overall, SSMG-LAMP achieved a sensitivity and specificity of up to 83.3 % (fluorescent detection) and 96.2 % (electrochemical detection), respectively. The highest accuracy was calculated to be 90.6 % in electrochemical measurement, primarily due to the low false-negative rate, while colorimetric and fluorescent detection methods accounted for 81.3 % and 84.4 % accuracy, respectively (Table S3). Further validation of these results (especially HPV 18+ samples) requires a larger number of clinical samples, however, as HPV 16 and 18 are responsible for 50–60 % and 10–15 % of cervical cancers respectively (Hidjo et al., 2024), the number of HPV 18+ samples can be significantly fewer.

While visual detection is ideal for resource-limited settings, however, this approach can compromise sensitivity and lead to false negatives, especially when the viral load is low. Fluorescent detection generally offers increased sensitivity, and in this case, the dual-color fluorescence enabled the detection of weak-positive samples. Rather than costly optics, this system used a fluorescent viewer that consisted of a battery-powered blue LED with a filter in a paper box, offering an affordable option to bring fluorescent detection in low-resource settings. While our results show that electrochemical detection can lower the rate of false negatives and there exist many commercial options of screen-printed electrodes that can be readily used, using electrochemical sensors for HPV screening in resource-limited areas can still be challenging. This is because performing electrochemical sensing requires specific equipment operated by personnel with relevant expertise. Yet, recent commercial electrochemical sensing systems gear toward palm-sized all-in-one platforms with USB or Bluetooth connections that can be operated on a smartphone. Overall, each detection strategy has its unique strengths and limitations; this system allows the use of strategies that are the most suitable in a given scenario.

## 4. Conclusion

This study presented a SSMG-LAMP assay for the rapid detection of high-risk HPV strains with a triple-mode signal readout with several advantages. First, this approach coupled a fluorescent and a chromogenic DNA intercalator in a LAMP assay, which enhances the color contrast during target detection, making the results distinguishable by the naked eye, even in low signal reactions. Second, the SSMG-LAMP assay enabled dual-color fluorescence, offering increased sensitivity compared with a single fluorescence readout. Third, this system is compatible with electrochemical sensing, which overcomes the limitations of colorimetric and fluorescent-based detection. Within 45 min, this SSMG-LAMP can detect as low as 10 copies μL^−1^ of HPV DNA and identify HPV 16 and 18 in unprocessed clinical urine samples. This proof-of-concept supports multiple modes of endpoint detection, which can be easily adapted to specific testing environments, which holds potential for HPV screening in resource-limited settings to reduce the disparity of cervical cancer burden, especially in low-income countries. While these experiments showed promising results, our future work will focus on the further validation and use of this platform for the pooled screening of multiple high-risk HPV subtypes in a large number of clinical samples. To a broader extent, the simplicity and versatility of this triple-mode detection system can facilitate the decentralization of a wide range of viral screening in multiple testing scenarios in resource-limited settings.

## Supplementary Material

SI

Appendix A. Supplementary data

Supplementary data to this article can be found online at https://doi.org/10.1016/j.biosx.2025.100658.

## Data Availability

Data will be published in the supplemental file
