# Supplementary material for: Triple-mode point-of-care diagnostics for high-risk human papillomavirus in urine
Source: Biosens Bioelectron X. Author manuscript; Available in PMC 2025 Aug 28. (PMC12377189; doi:10.1016/j.biosx.2025.100658)
Supplement: SI [file NIHMS2105777-supplement-SI.docx]

**Supplementary data**

**Triple-mode point-of-care diagnostics for high-risk human papillomavirus in urine**

Duc Anh Thai ^a,b^, Jing Liu ^a,b^, Angel Gutierrez Ruiz ^a,c^, Yuqian Zhang ^a,b^, Marina Walther-Antonio ^b,d,e^, Yuguang Liu ^a,b,e,f, *^

*^a^ Department of Physiology and Biomedical Engineering, Mayo Clinic, Rochester, MN 55905, USA*

*^b^ Microbiome Program, Center for Individualized Medicine, Mayo Clinic, Rochester, MN 55905, USA*

*^c^ Department of Chemistry and Biochemistry, The University of Texas at El Paso, El Paso, TX 79968, USA*

*^d^ Department of Obstetrics and Gynecology, Mayo Clinic, Rochester, MN 55905, USA*

*^e^ Department of Surgery, Mayo Clinic, Rochester, MN 55905, USA*

*^f^ Department of Immunology, Mayo Clinic, Rochester, MN 55905, USA*

*^*^ E-mail:* [*Liu.Yuguang@mayo.edu*](mailto:Liu.Yuguang@mayo.edu) *(Yuguang Liu)*


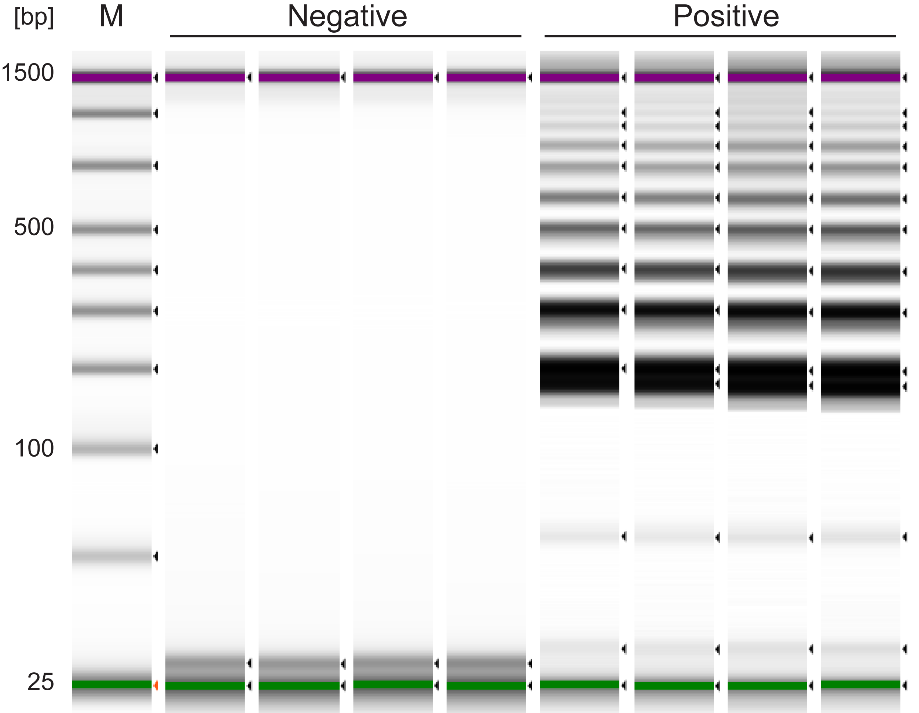


**Fig. S1.** Electrophoresis analysis of LAMP products. The reaction was performed with HPV 16 primer set at 65 °C for 30 min. M: DNA ladder, Negative control with no DNA template and Positive sample with 1200 copies µL^−1^ of HPV 16 DNA.


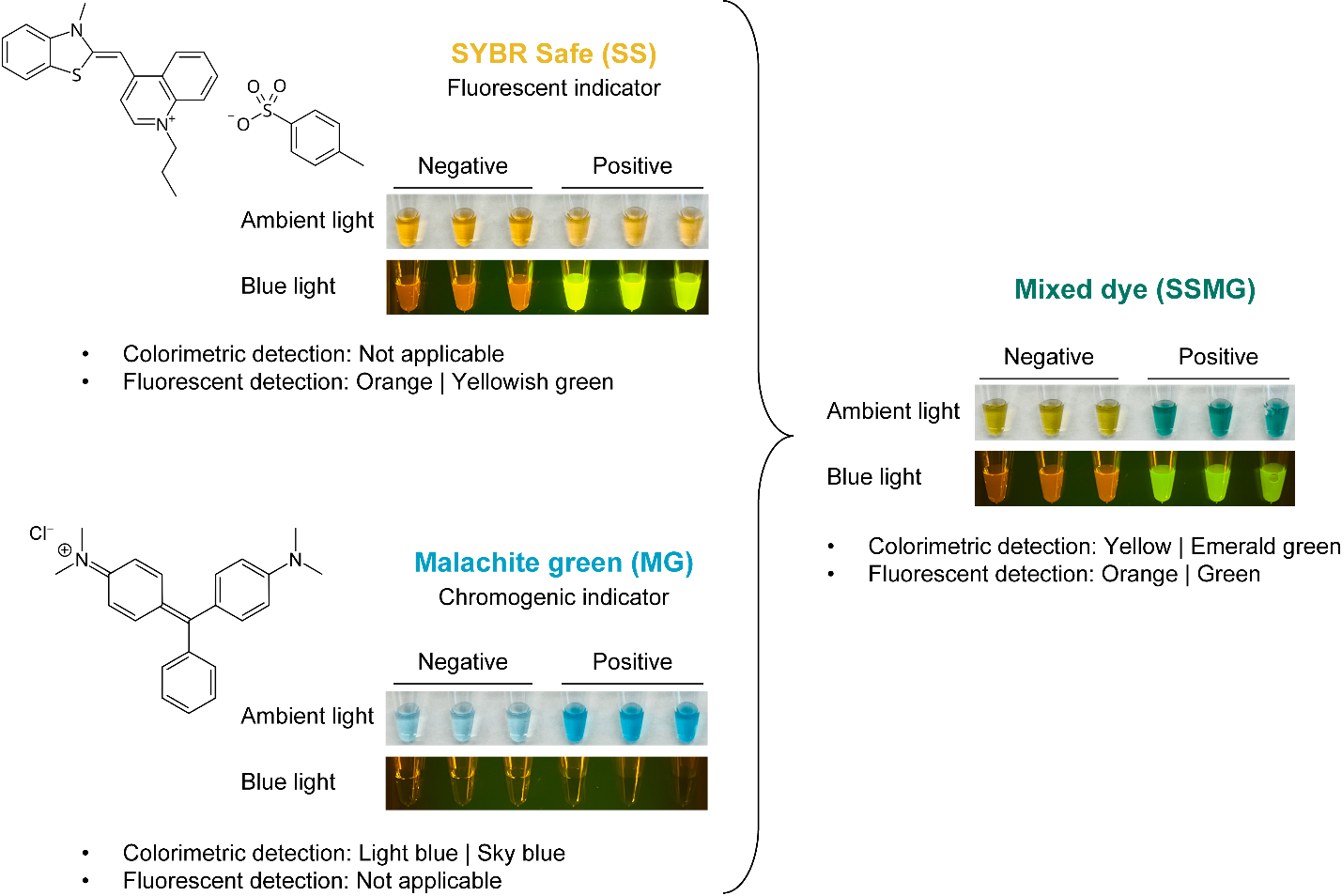


**Fig. S2.** Colorimetric and fluorescent LAMP detection using SYBR Safe, malachite green, and mixed dye. Negative control with no DNA template and Positive sample with 1200 copies µL^−1^ of HPV 16 DNA.


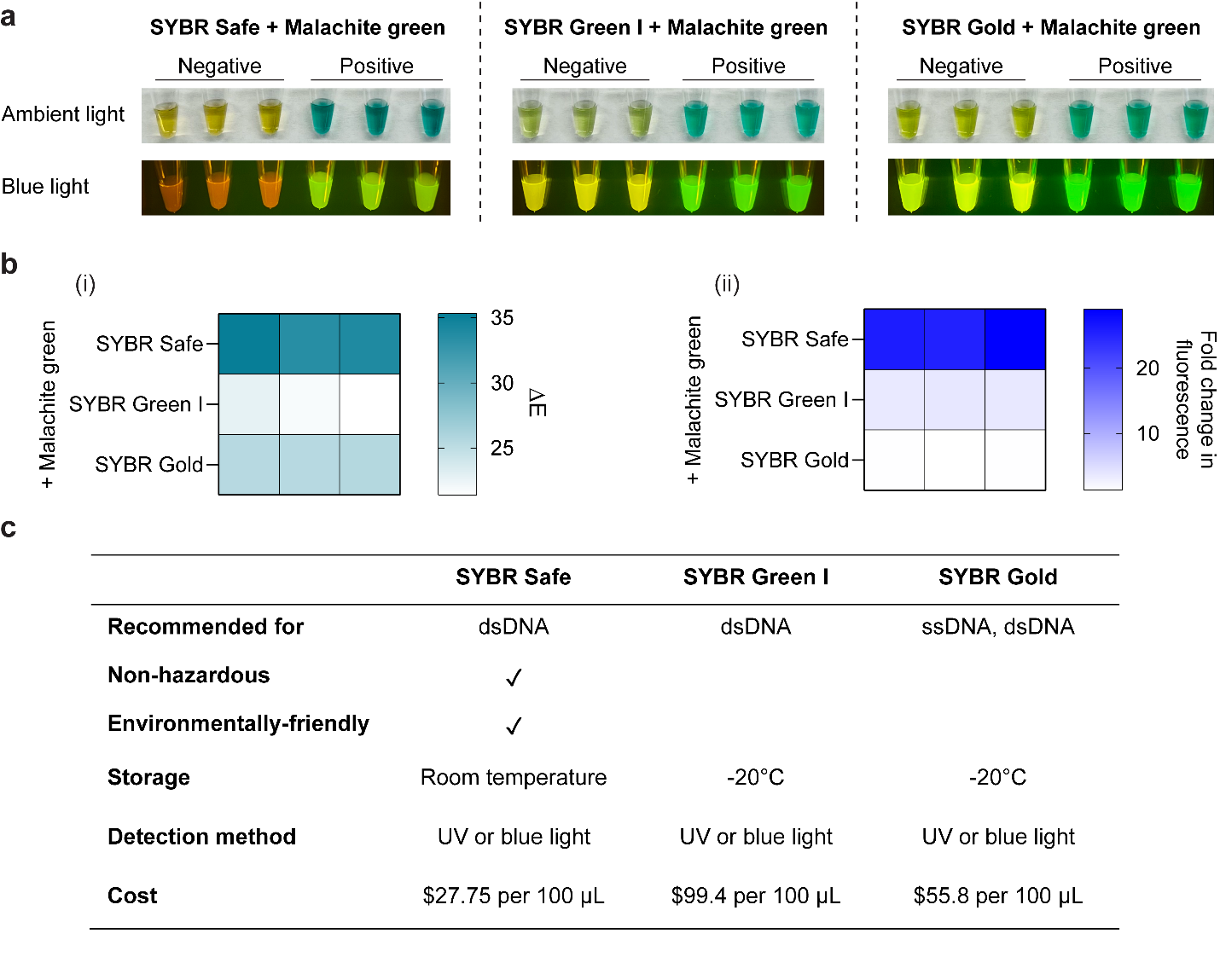


**Fig. S3.** Effect of different SYBR dyes on the assay’s performance. (a) Colorimetric and fluorescent visualization. (b) Heat maps of (i) color difference (∆E) between the HPV 16+ sample and the negative control and (ii) fold change in fluorescence of the HPV 16+ sample over the negative control. (c) Comparison of the SYBR family of DNA intercalating dyes, based on Thermo Fisher Scientific Inc.’s guide. The reaction was performed with HPV 16 primer set at 65 °C for 30 min. M: DNA ladder, Negative control with no DNA template and Positive sample with 1200 copies µL^−1^ of HPV 16 DNA.


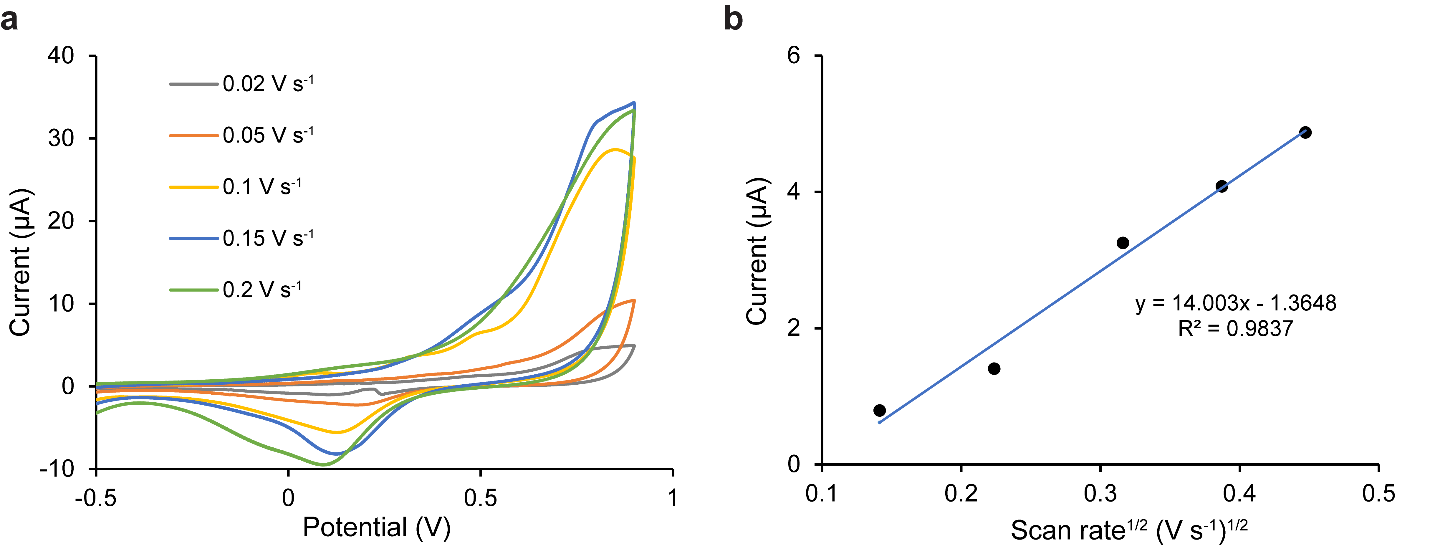


**Fig. S4.** Effect of scan rate on cyclic voltammetry in electrochemical measurement. (a) Cyclic voltammetry obtained at various scan rates (0.02 – 0.2 V s^−1^) in SSMG-LAMP solution. (b) Plot of cathodic peak current versus the square root of scan rates.


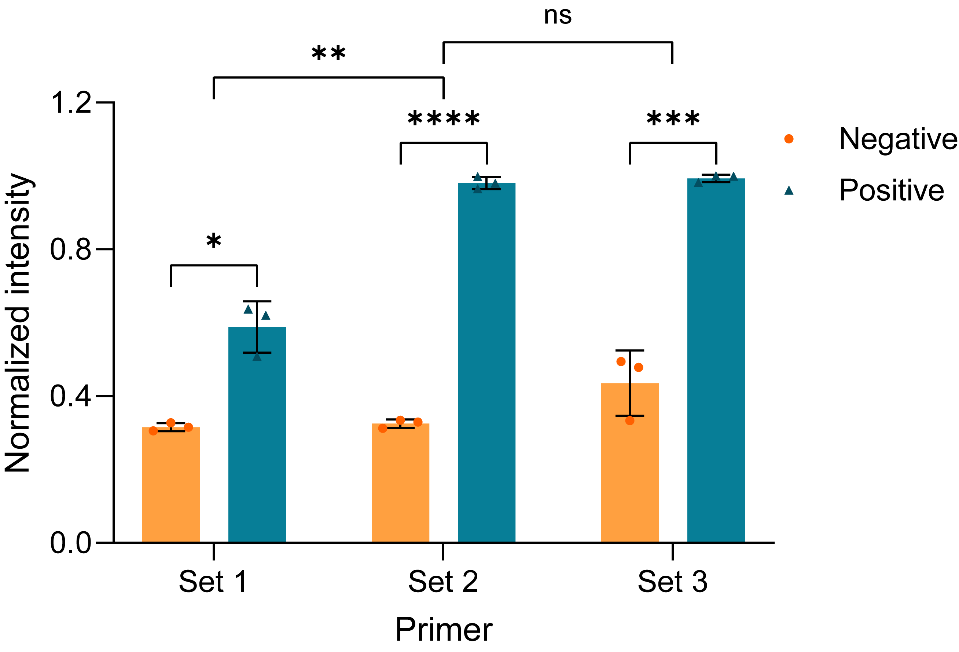


**Fig. S5.** Effect of primer concentrations on SSMG-LAMP assay. Primer Set 1: 0.1 μM for F3/B3 primers, 0.8 μM for FIP/BIP primers, and 0.2 μM for LF/LB primers; Primer Set 2: 0.2 μM for F3/B3 primers, 1.6 μM for FIP/BIP primers, and 0.4 μM for LF/LB primers; Primer Set 3: 0.4 μM for F3/B3 primers, 3.2 μM for FIP/BIP primers, and 0.8 μM for LF/LB primers. Negative control with no DNA template and Positive sample with 1200 copies µL^−1^ of HPV 16 DNA. ns: *p* > 0.05, **p* ≤ 0.05, ***p* ≤ 0.01, ****p* ≤ 0.001, *****p* ≤ 0.0001.


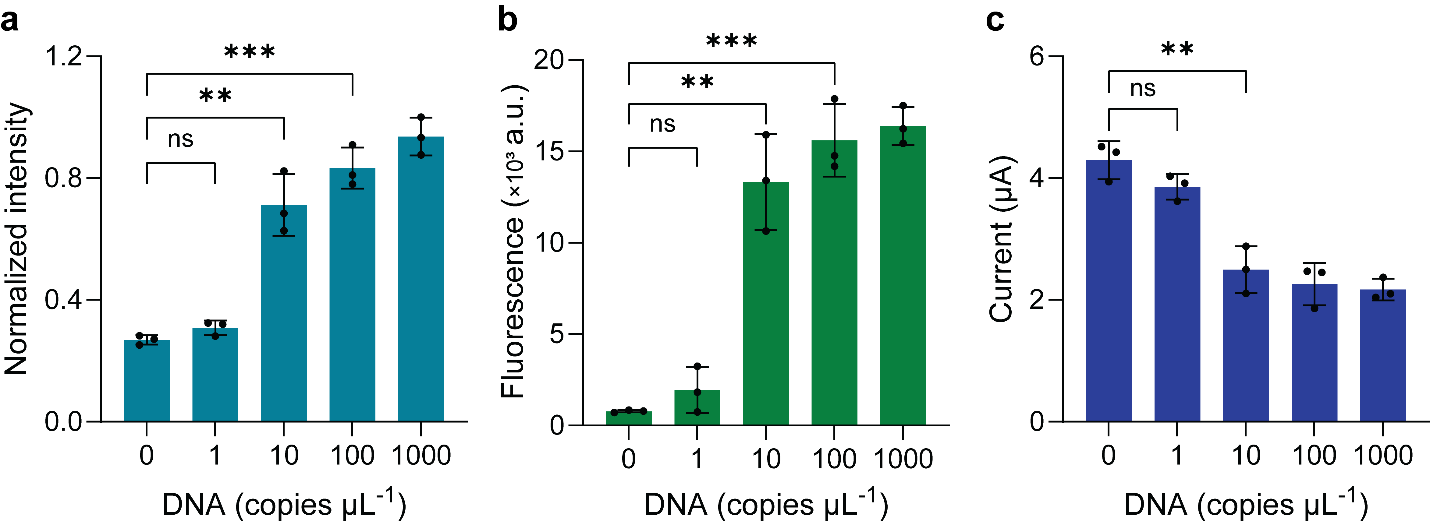


**Fig. S6.** Sensitivity test of SSMG-LAMP assay for HPV 18 detection. Colorimetric (a), fluorescent (b), and electrochemical (c) signals of SSMG-LAMP at different HPV 18 DNA concentrations. ns: *p* > 0.05, ***p* ≤ 0.01, ****p* ≤ 0.001. The experiment was performed in three replicates.


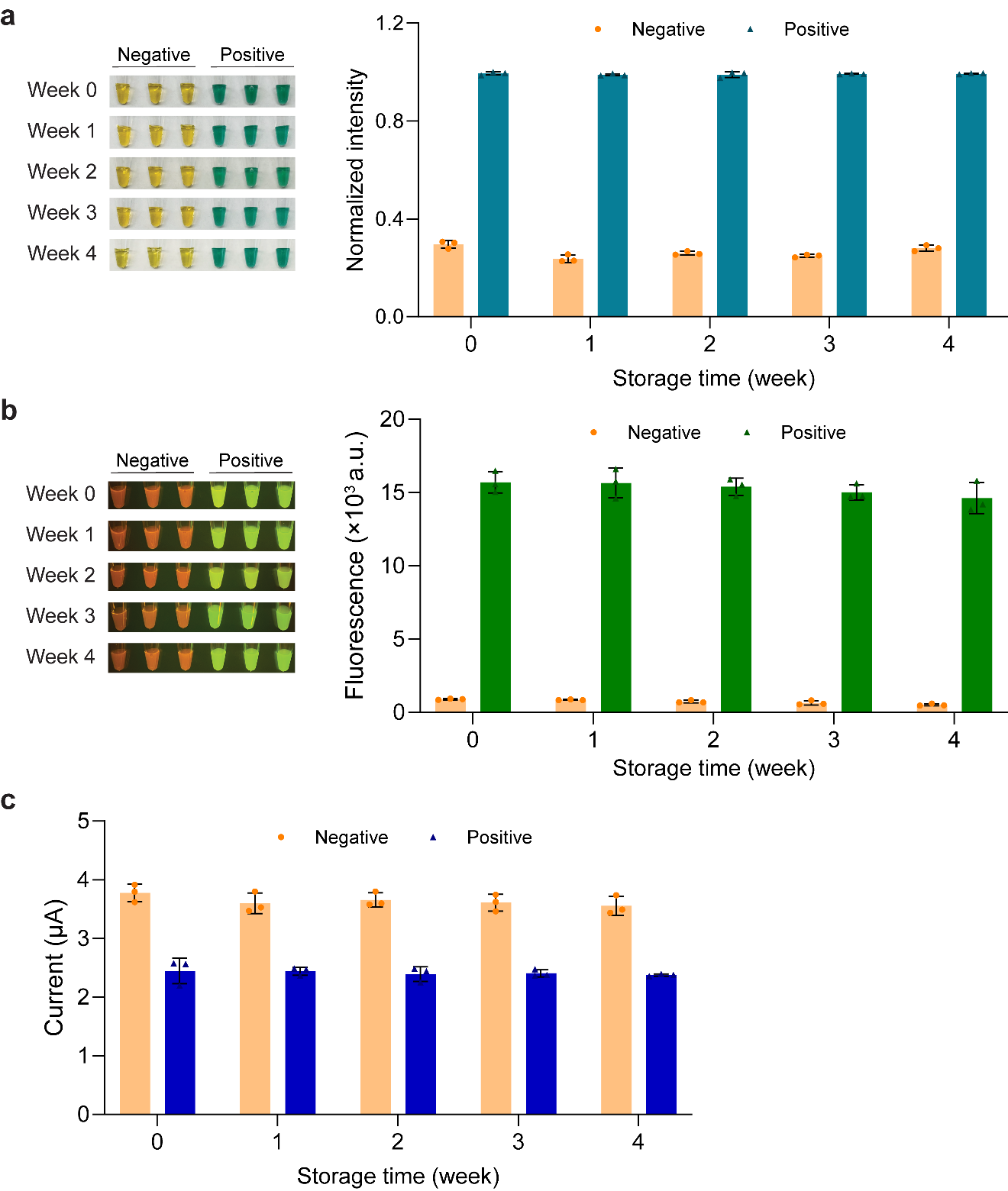


**Fig. S7.** Stability of SSMG for HPV detection. (a) Visual detection and color intensity, (b) fluorescent visualization and intensity, and (c) electrochemical measurement of SSMG-LAMP assay toward 4-week storage at room temperature. Negative control with no DNA template and Positive sample with 1200 copies µL^−1^ of HPV 16 DNA.

**Table S1.** Sequences of LAMP primers for amplifying *E6* gene of HPV 16 and 18.

| **Target** | **Primer** | **Sequence 5'-3'** |
| --- | --- | --- |
| **HPV 16** | F3 | ATGCACCAAAAGAGAACTG |
|  | B3 | AGCATATGGATTCCCATCTC |
|  | FIP | GCAGCTCTGTGCATAACTGTCAATGTTTCAGGACCCACA |
|  | BIP | AGAATGTGTGTACTGCAAGCAATCCCGAAAAGCAAAGTCAT |
|  | LF | GGTAACTTTCTGGGTCGCTCC |
|  | LB | CAGTTACTGCGACGTGAGGT |
| **HPV 18** | F3 | AAAAACTAACTAACACTGGGTTA |
|  | B3 | ACTTGTGTTTCTCTGCGT |
|  | FIP | GGTGTCTAAGTTTTTCTGCTGGATAATTTATTAATAAGGTGCCTGCG |
|  | BIP | CGACGATTTCACAACATAGCTGGGTTGGAGTCGTTCCTGTC |
|  | LB | ATAGAGGCCAGTGCCATTCGTG |

*F3:* forward outer primer*; B3:* backward outer primer*; FIP:* forward inner primer*; BIP:* backward inner primer*; LF:* forward loop primer*; LB:* backward loop primer*.*

**Table S2**. Information of the 16 clinical urine samples tested in this study.

| **Sample** | **Collecting label** | **HPV 16** | | | | **HPV 18** | | | |
| --- | --- | --- | --- | --- | --- | --- | --- | --- | --- |
|  |  | Clinic | Colorimetry | Fluorometry | Electrochemistry | Clinic | Colorimetry | Fluorometry | Electrochemistry |
| S1 | 1001-U | - | - | - | - | - | - | - | - |
| S2 | 1002-U* | - | - | - | - | - | - | - | - |
| S3 | 1003-U* | - | + | + | - | - | - | - | - |
| S4 | 1004-U* | - | - | - | - | - | - | - | - |
| S5 | 1005-U | - | + | + | - | - | - | - | - |
| S6 | 1006-U* | - | + | + | + | - | - | - | - |
| S7 | 1007-U | - | - | - | - | - | - | - | - |
| S8 | 1008-U | + | + | + | + | - | - | - | - |
| S9 | 1011-U | - | - | - | - | + | - | + | - |
| S10 | 1013-U* | - | - | - | - | - | - | - | - |
| S11 | 1014-U* | + | + | + | - | - | - | - | - |
| S12 | 1025-U* | + | + | + | + | - | - | - | - |
| S13 | 1032-U | - | - | - | - | - | - | - | - |
| S14 | 1036-U | + | + | + | + | - | - | - | - |
| S15 | 1044-U | + | - | - | + | - | - | - | - |
| S16 | 1051-U* | - | + | + | - | - | - | - | - |

***Other HR-HPV positive

**Table S3**. Sensitivity, specificity, and accuracy of SSMG-LAMP assay for HPV 16 and 18 detections in clinical urine samples.

|  |  | **Clinic** | |  |
| --- | --- | --- | --- | --- |
|  |  | Positive | Negative |  |
| **Colorimetry** | Positive | 4 | 4 |  |
|  | Negative | 2 | 22 |  |
|  |  | Sensitivity (66.7 %) | Specificity (84.6 %) | Accuracy (81.3 %) |
| **Fluorometry** | Positive | 5 | 4 |  |
|  | Negative | 1 | 22 |  |
|  |  | Sensitivity (83.3 %) | Specificity (84.6 %) | Accuracy (84.4 %) |
| **Electrochemistry** | Positive | 4 | 1 |  |
|  | Negative | 2 | 25 |  |
|  |  | Sensitivity (66.7 %) | Specificity (96.2 %) | Accuracy (90.6 %) |
